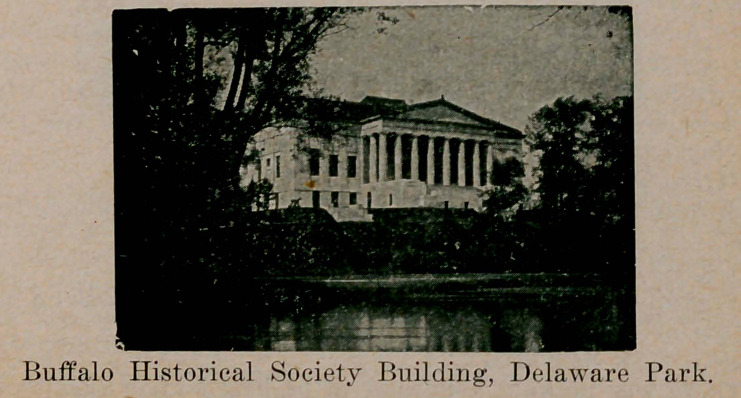# Anemia: An Undescribed Cause: Its Easy Detection by the General Practitioner

**Published:** 1915-04

**Authors:** Pierce J. Candee

**Affiliations:** 502 Normal Avenue


### Anemia: An Undescribed Cause: Its Easy Detection By the General Practitioner.

By PIERCE J. CANDEE, M. D.,

Attending Physician Sisters of Charity and St. Mary's Hospitals; Member of N. Y. State Society; Buffalo Academy of Medicine.

To go deeply into the subject of anemia would be far beyond the scope of one paper, in fact, it would be rather trying to the patience of a reader or the essayist. But two or three important points regarding this condition the standard works make little or no mention of: I refer to the derevation of hemoglobin, the cause when not supplied in sufficient quantities and a test for anemia that is almost equal to, if not fully as positive as a blood count or other tests that require expensive apparatus and a fineness of technic.

We are all aware, even high school students are taught this, the brain, muscles, bones, and blood have a specific food which is obtained from the food we consume being utilized through a process of digestion and assimilation. It therefore must be very plain deductions, if these different substances are to be supplied in normal amounts; food, and food of a nutritious character must be furnished—also there must be perfect digestion and assimilation. It may be admitted that food may be supplied in sufficient quantities and digestion and assimilation perfect, still marked anemia be present. But in these cases we have a well-defined cause, a condition described fully in any work on the blood and which can be stated as follows (Cabot):

1. Infective or febrile diseases, acute or chronic.
2. Malignant disease.
3. Chronic suppurations, nephritis and dysentery, cirrhosis of the liver.
4. Bad hygiene, pregnancy and lactation.
5. Intestinal parasites, poisoning by lead, arsenic, etc.

In all these conditions the average physician is fairly well versed. He realizes that in typhoid, suppurations, Bright's drains on the system and the various conditions mentioned there is anemia to a more or less degree, he can see it (too much dependence must not be placed on the appearance of the conjunctiva, nails, lips, and skin) and if necessary can partially prove it by the hemoglobin scale. But it is in conditions where the above causes are not present, except as the result from the condition to be alluded to, that attention is to be drawn. For example, it is the growing active young adult, or for that matter, the matured one, the one reared under the best of environments; when they develop anemia (usually the last condition thought of) what is the cause, what is the remedy?

It has already been stated, hemoglobin is obtained from the food by a process of digestion and assimilation: if it is insufficiently supplied, yet food is taken in normal quantities, the cause is very clear, digestion and assimilation are defective. The solution of this is very plain.

The nervous system controls digestion and assimilation: If this system is in an imperfect condition it cannot carry on its functions in a normal manner, thus we have impairment of the aforesaid. But let us go a step further, the nervous system has a food, a food that must be taken from the food consumed, and if this is limited from any reason, this system must suffer also: thus it is found that not only nerve nourishment, but hemoglobin is insufficiently supplied and neuroses and anemia result.

The causes of impairment of the nervous system, the condition that must be looked to first are so numerous that unlimited space would be necessary in their mention. Suffice it to say, however, anything and everything making an impression, mental in nature, influences the nervous system and calls for energy. To be more explicit, anything thought, word or deed, calls for mental activity, and in doing so energy must be used; energy that is furnished solely by the action of the brain, the great central storage battery.

One other condition in connection with the nervous system, as a source of energy, is the fact that the nerve-cells have a reserve that can be called upon in time of stress, when from any reason food is not taken per mouth in sufficient quantities to meet the daily demand. We as physicians are fully aware of one thing. a goodly proportion of humanity overtax their nervous systems; energy is used beyond that normally supplied and the reserve must be called on. If the deficiency is replaced by having perfect digestion and assimilation, together with mental rest, the great rejuvenator, all is well, but if vice-versa sooner or later depletion takes place to an extent that normal action in the different organs is evidenced. The gastro-intestinal track suffers among the very first; there is indigestion and malassimilation, hemoglobin is insufficiently supplied and we have anemia as a result.

The tests of anemia are simple, yet if any positiveness is obtained certain technic and quite a costly apparatus is necessary. A microscope and Thoma-Zeiss counting slide, if cells are to be counted or viewed—a Von Fleischel's hemometer, the only positive test for hemoglobin, for although the hemoglobin scale is simple and inexpensive, the information obtained is very vague, and cannot be depended upon.

As to visual examination of the patient; conjunctiva, nails, lips or skin a diagnosis by this means is little more than a guess: In 189 females who looked anemic, Dr. Stone made careful blood examinations and found 75 per cent. in 89 (75 per cent. hemoglobin in the female is normal) or about one-half had a normal amount.

It may be briefly stated that in at least 50 per cent. of cases where iron is prescribed, from appearances, it is not necessary; in fact is contra-indicated. But one other point of valuable information may be stated here; if the system is unable to take sufficient hemoglobin from the food, on account of inefficient action on the part of the digestive and assimilative processes, how can an artificially supplied substitute be absorbed?

Fully 95 per cent. of office patients do not want to wait for technical examinations, many cannot afford to pay for such, appearances are deceptive yet these people should be prescribed for with every possible degree of accuracy as to their condition. The method of ascertaining anemia, and one as already stated fully as positive as a blood count, is the finding of albumin (generally a faint trace) in the urine.

Of course, other causes of albumin should be eliminated, yet this can be easily and quickly done in the course of an office examination. Although the causes of Albumin are fairly well-known they may be briefly referred to, and in order of frequency as follows:

1. Disturbances or disease of the urinary track below the kidneys; such means any inflammation, thus we find albumin often present in gonorrhoeal cases: always in pyelitis, and if substances from the genital track enter the urine, spermatozoa.

2. Changes in the kidney structure, Bright's Disease.

3. Abnormal changes in the quality of the blood, the serum albumin being rendered more diffusable. This condition under which anemia occurs is supposed to be due to a lessened nutrition of the renal cells.

4. Alterations in the blood pressure of the kidneys, physiological or functional albuminuria, found in soldiers after a prolonged march; sudden chilling of the body and occasionally in neurotic subjects.

In examining for urine, when using the nitric acid test, one thought should always be foremost in the examiners mind; the stimulating diuretics as oil of sandal wood, copaiba, cubebs, will give to the urine a whitish tint that may be mistaken for albumin.

In speaking of anemia, Cabot says, "In one sense all anemias are secondary; it is due to some cause, a symptom is a change of events. But in some cases we know the cause and in some we do not." Take for instance the disease chlorosis, it is known to be secondary anemia and is divided into four stages.

1st there is no diminution in the red cells, but merely in the amount of cellular substance, hemoglobin. 2nd practically the same. 3rd red cells begin to suffer, and 4th there is degeneration and destruction of the cells. Further, Cabot says, "There are no evidences of primary diseases of the blood-making functions." With this statement, and the fact that in practically the fourth stage of chlorosis is reached there is no diminution in cells, and the further fact that all chlorotics are of a highly neurotic temperament, is it not fair to assume the blood is being deprived of its normal nutrition by not being able to obtain the same in a physiological manner; and the cause, is it not plainly evident is due to inefficient action on the part of the digestive and assimilative functions which are entirely under control of the nervous system. But chlorosis is only one example, are not the great majority of cases where a well-defined cause (see causes) is evident due to the same condition?

Miss K., age 14. This young maiden was seen in consultation giving a history of menses coming on one year previously, but had occurred only every other month and was scanty at that. She could not keep up with her studies; it was thought she would have to leave school. Her physical appearance was fairly good; she was tall, well formed but not very robust. There was an anemic look (hemoglobin 66 per cent.) the characteristic history of eating slate pencils, pickles and other indiscretions familiar to all. There was a good family history back to the third generation, with the possible exception of neuroses.. Careful examination of the urine revealed nothing wrong, except a faint trace of albumin and an index 85 per cent minus. Comp. Phos. Tonic (Dowd)* 10 to 15 minims in milk about 30 to 40 minutes after meals. Briefly speaking the following result was obtained: menses the next month increased fully 50 per cent, in three months she was regular, and for 4 days. Facial appearances changed decidedly, she gained in flesh became robust and in six months was enjoying the best of health, menstruating regularly for four days, not tireing at the least exertion and finding it no trouble to keep up with her studies at school. Index now 10 per cent. minus.

Briefly summed up the following conclusions can be arrived at:

1. Where well defined causes are absent, (tables referred to) and which is true in a goodly number of cases, digestive and assimilative processes should be looked to.

2. These being entirely under control of the nervous system,

* This is a mixture of nux vomica, ignatia, cannabis indica and elementary phosphorus which is easily proven by the fact it smokes when dropped in water and becomes luminous in the dark. In several cases where nux vomica, iron and other drugs have failed to give any result this mixture has been substituted with the most gratifying results, the effect was immediate and permanent.

ascertain the condition of this system by aid of the phosphatometer.

3. A minus index means insufficient nerve nourishment, and calls for nerve foods, chief of which is phosphorus.

4. When albumin is found in the urine, and the well known causes can be eliminated (Illumination of the urinary track and Bright's disease) in fully 95 per cent. of the cases it is due to anemia and in at least 75 per cent. of these conditions due to nerve cell starvation and will be easily and quickly relieved by nerve food.

502 Normal Avenue.

## Figures and Tables

**Figure f1:**
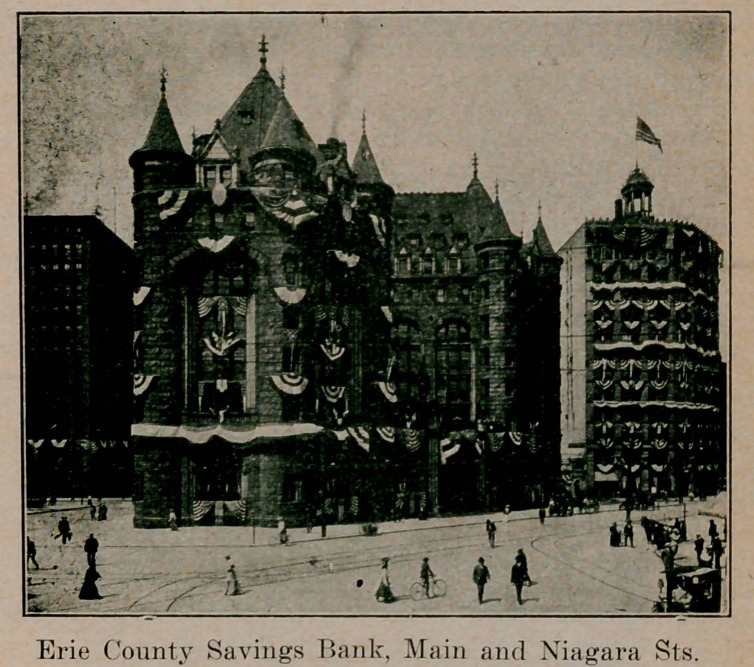


**Figure f2:**